# Surgical Site Infection in Coronary Artery Bypass: Observational Study

**DOI:** 10.1111/ijn.70137

**Published:** 2026-03-25

**Authors:** Camila Vieira Gebhardt, Gabriel Lopes Vieira Silva, Amanda Gubert Pereira, Clesnan Mendes Rodrigues, Valéria Nasser Figueiredo, Maria Beatriz Guimarães Raponi

**Affiliations:** ^1^ Obstetric Nursing, Hospital Sofia Feldman Belo Horizonte Minas Gerais Brazil; ^2^ Faculty of Medicine Federal University of Uberlândia Uberlândia Minas Gerais Brazil; ^3^ Federal University of Triângulo Mineiro Uberaba MG Brazil

**Keywords:** cardiac surgical procedures, coronary artery bypass, infection control, perioperative care, surgical wound infection

## Abstract

**Background:**

Preventing surgical site infections plays a crucial role in ensuring patient safety during coronary artery bypass grafting. To reduce incidence rates, it is essential to implement evidence‐based best practice measures. However, there is a noticeable lack of studies assessing healthcare professionals' adherence to these measures, which justifies the present investigation.

**Aim:**

This study aimed to determine adherence to surgical site infection prevention measures, determine the incidence of surgical site infection and examine the relationship between adherence and infections in patients undergoing coronary artery bypass grafting.

**Methods:**

This was an observational, longitudinal study with a quantitative approach, conducted at a large university hospital, recognized as a regional referral centre for cardiac procedures. A nonprobabilistic recruitment process was employed, and no selection bias was identified. All patients scheduled for elective coronary artery bypass grafting during the study period from September 2022 to May 2023 were included.

**Results:**

The mean adherence score to the recommended practices was 71.18%. Among the participants, 24 (55.8%) developed infections, of which 10 (23.3%) required hospital readmission and 3 (7.0%) resulted in death.

**Conclusions:**

Despite a relatively high adherence to preventive measures, the incidence of surgical site infections was also significant (55.8%), underscoring the urgent need for more effective prevention strategies in coronary artery bypass grafting.

## Abstract (highlights)

**What Is Already Known About This Topic?:**

- Preventing surgical site infections plays a crucial role in ensuring patient safety during coronary artery bypass grafting (CABG).
- To reduce incidence of surgical site infections, it is essential to implement evidence‐based best practice measures.
- However, there is a noticeable lack of studies assessing healthcare professionals' adherence to these measures.

**What This Paper Adds?:**

- A high incidence of infection (55.8%) in a population of patients is undergoing CABG.
- There was an average adherence of 71.18% to preventive measures, highlighting a gap between practice and established guidelines.
- A clinically relevant association where each additional percentage point in adherence to preventive measures was linked to a 10% reduction in the odds of infection.

**The Implications of This Paper:**

- Despite a relatively high adherence to recommended practices, the incidence of surgical site infections was significant, suggesting that failures at critical points in clinical practice may be a determining factor.

## 1 Introduction

Surgical site infections (SSIs) are common and clinically significant complications in patients undergoing coronary artery bypass grafting (CABG), often leading to adverse outcomes (Hadaya et al. 2022). It is estimated that patients who develop infectious complications following CABG incur an additional hospitalization cost of R$ 37007.95 within the Brazilian Unified Health System (SUS) (Barbosa et al. 2019).

SSIs rank third among healthcare‐associated infections (HAIs), accounting for approximately 14%–16% of infections observed in hospitalized patients (Brazil 2017). An integrative review covering the period from 2011 to 2021 analysed 24 studies, which reported SSI incidence rates following CABG ranging from 1.4% to 38% and mortality rates between 2.4% and 38.9% (Fiorin et al. 2022). Notably, the risk of mortality is significantly higher in patients who develop SSI compared to those who do not, and this disparity is even more pronounced in developing countries due to limited access to essential treatments and healthcare resources (Kaspersen et al. 2021; Barbosa et al. 2019).

SSIs significantly impact patients' quality of life, often resulting in decreased vitality, increased pain intensity and reduced ability to perform physical activities and daily functions (Hart et al. 2021). The need for additional treatments due to infection contributes to prolonged hospitalization and delayed postoperative recovery, which in turn leads to emotional distress and adverse effects on mental health (Totty et al. 2021).

Several risk factors contribute to the development of SSIs, which include both intrinsic and extrinsic factors. Among the intrinsic patient‐related factors, advanced age, male sex, diabetes mellitus (DM), chronic kidney disease (CKD), arterial hypertension, obesity and prior colonization by multidrug‐resistant pathogens are notable (Bustamante‐Munguira et al. 2019). Extrinsic factors are associated with prolonged surgical duration and cardiopulmonary bypass (CPB), as well as poor adherence to preventive measures such as hand hygiene and early antimicrobial prophylaxis (Silva and Damasceno 2020; Kanasiro et al. 2019).

Prevention plays a fundamental role in reducing the incidence of SSIs. Evidence‐based guidelines, such as those issued by the World Health Organization (2016) and the Centers for Disease Control and Prevention (Berríos‐Torres et al. 2017), recommend the adoption of a standardized set of interventions, including antimicrobial prophylaxis, maintenance of normothermia, glycemic control, and strict aseptic technique, to prevent SSIs in procedures such as CABG. Additionally, the implementation of care bundles and perioperative safety checklists has been shown to improve adherence to preventive measures and is associated with reduced SSI rates (Gakuanyi et al. 2022; Cussotto et al. 2025). There is a noticeable scarcity of studies in the literature evaluating healthcare professionals' adherence to these measures, which justifies the development of the present study (Deslarzes et al. 2023; de Oliveira and Gama 2015).

## 2 Methods

### 2.1 Aim and Design

The aim of this study was (1) to determine adherence to SSI prevention measures and the incidence of SSI and (2) to examine the relationship between adherence and SSI in patients undergoing coronary artery bypass grafting.

This was an observational, longitudinal study with a quantitative approach, conducted at a large university hospital with 520 beds, recognized as a regional referral centre for cardiac procedures, located in Uberlândia, Minas Gerais, Brazil.

### 2.2 Participants and Sample Definition

The study included patients aged 18 years or older who underwent elective coronary artery bypass grafting. Patients who underwent emergency surgeries or had preexisting infections were excluded from the study.

The sample size calculation was based on a moderate negative linear correlation of *r* = 0.4 between total adherence score and length of hospital stay, assuming a significance level (alpha) of 0.05 and a statistical power of 80%. Accordingly, the minimum required sample size was *n* = 46. A nonprobabilistic recruitment process was employed, and no selection bias was identified. All patients scheduled for elective CABG during the study period from September 2022 to May 2023 were included. During this period, a total of 58 patients were identified. This study considered a nonprobabilistic and sequential sample of all patients scheduled during the described period.

### 2.3 Data Collection

Data were collected using a structured instrument composed of three sections: Sociodemographic and Clinical Characteristics, Perioperative Safety Checklist and Incidence of Postoperative SSI.

The Sociodemographic and Clinical Characteristics section included the following variables: sex, age, body mass index (BMI), preexisting conditions, unhealty lifestyle habits, history of previous cardiac surgery, presence and type of prostheses or implants, use of drains, preoperative blood transfusion, patient origin, American Society of Anesthesiologists (ASA) score, length of hospital stay, length of ward stay prior to surgery, operating room temperature, duration of surgery, duration of anaesthesia, type of anaesthesia, use of CPB, CPB duration, intraoperative hypothermia during CPB, hypothermia duration and preoperative and intraoperative laboratory tests (platelet count, haemoglobin and haematocrit).

The Perioperative Safety Checklist is a validated tool designed to assess adherence to best practices during the perioperative period. It consists of 48 questions addressing key practices for the prevention of infections in surgical settings. The checklist is divided into five stages: admission to the operating room, prior to anaesthesia induction and surgical draping, before surgical incision, before leaving the operating room and before discharge from the surgical centre (Roscani et al. 2015).

The final section, Incidence of Postoperative SSI, involved data on SSI occurrence. The definition of SSI adopted was based on specific national criteria established by the National Health Surveillance Agency (ANVISA) (Brazil 2017), a Brazilian regulatory agency linked to the Ministry of Health; these are infections related to surgical procedures in hospitalized or outpatient patients, with or without implants, affecting the incision, tissues, organs or cavities involved and are classified as superficial incisional, deep incisional or organ/space, occurring within the first 30 days after surgery or up to 90 days. In this study, all SSIs related to the surgical procedure, diagnosed during follow‐up up to 90 days, were included. This section includes items related to infection presence, need for hospital readmission due to infection, infection site, onset and timing of symptoms, use of antimicrobials, wound characteristics and identification of microorganisms.

Data collection was conducted by two researchers who previously carried out a pilot study for training purposes, including data collection and database completion with preliminary statistical analysis. The process began with monitoring the scheduled surgeries through the hospital's Information System, which provided the expected date and time of the procedure, along with the patient's full name, date of birth, and medical record number.

On the day of the procedure, either in the inpatient ward or upon admission, the researchers made the first contact with the patient to assess inclusion and exclusion criteria, determine eligibility, obtain Informed Consent Form and collect data for the first section of the instrument based on self‐reported responses and medical record information.

For data collection of the second section, the researchers waited for the participant's admission to the operating room, where axillary temperature was measured using a TH400 G‐tech thermometer. Inside the operating room, the researchers positioned themselves strategically to observe and collect data without disrupting workflow. Glycemic control was performed by the anesthesiologist through blood samples drawn from the central venous access and measured with a glucometer at various intraoperative time points. All participants had their core body temperature continuously monitored intraoperatively using a skin temperature sensor connected to a DX2023 Dixtal multiparameter monitor. Measurements were recorded by the perfusionist every 5 min along with CPB data. When necessary, the researchers communicated with the surgical team and reviewed intraoperative documentation to verify information.

Finally, for the third section of the instrument, participant follow‐up was conducted through active surveillance of electronic medical records for up to 90 days postsurgery to identify the occurrence of SSI. This information was collected from clinical progress notes recorded in the medical record. In parallel, electronic records from the hospital Infection Prevention and Control (IPC) Committee were accessed to obtain complementary data. All infection‐related information was extracted from medical records and confirmed by the IPC medical team.

### 2.4 Ethical Considerations

Ethical approval was obtained, and all participants signed the Informed Consent Form, and the privacy and confidentiality of the subjects and the confidential data involved in the research were maintained.

### 2.5 Data Analysis

Data were analysed using IBM Statistical Package for the Social Sciences (SPSS) Statistics for Windows, version 23.0. Absolute and relative frequency distributions were used for categorical variables, while measures of central tendency (mean and median) and variability (standard deviation) were used for quantitative variables. Adherence to the recommended measures was assessed by counting positive responses (‘yes’) scored as 1 (one) for each item on the checklist, according to the following formula: Overall adherence = (Sum of positive items adherence/Total number of completed items) × 100.

To determine the incidence of SSI, the formula provided by ANVISA was applied: (Total number of SSIs related to the surgical procedure during the period/Total number of surgical procedures performed during the period) × 100 (Brazil 2017).

To analyse the influence of the total adherence score, which was considered the predictor variable because it was assessed prior to the occurrence of infection, on SSI, a preliminary binomial logistic regression analysis was performed including only one predictor, considering the limited sample size of this study. Therefore, the prerequisites for using this model with a quantitative predictor were appropriately considered, particularly the Hosmer–Lemeshow goodness of fit test.

## 3 Results

Of the 58 patients, 43 were included and 15 were excluded (cancellation rate of 24.1%). Among the exclusions, 14 surgeries were cancelled due to a healthcare professionals' strike at the institution, and in one case, the researchers were unable to remain in the operating room at the request of the medical team because of the high number of professionals present, which posed a consequent infection risk.

Among the 43 patients observed, there was a predominance of males (83.7%), with a mean age of 62.35 years (SD = 10.68), ranging from 34 to 84 years. Regarding nutritional status, the mean weight was 74.11 kg (SD = 13.80), ranging from 41.6 to 110 kg, and the mean height was 1.66 m (SD = 0.07), ranging from 1.48 to 1.79 m.

Table 1 presents the sociodemographic and clinical characteristics of the participants evaluated in the study.

**TABLE 1:** Sociodemographic and clinical characteristics of patients undergoing coronary artery bypass grafting (*N* = 43; Uberlândia, MG, Brazil, 2022–2023).

Variables	*N* ^a^	%^b^
Preexisting conditions		
Arterial hypertension	36	83.7
Diabetes mellitus	22	51.2
Dyslipidemia	11	25.6
Obesity	08	18.6
Chronic kidney disease	03	7.0
Chronic obstructive pulmonary disease	02	4.7
Unhealthy lifestyle habits		
Tobacco use	16	37.2
Alcohol use disorder	15	34.9
None	12	27.9
Blood transfusion		
No	38	88.4
Yes	05	11.6
Admission source		
Home	14	32.6
Emergency department	11	25.6
Inpatient ward	10	23.3
Intensive care unit	05	11.6
Other institution	03	7.0
ASA score		
III	41	95.3
II	01	2.3
IV	01	2.3

^a^ *N* = number.

^b^ % = percentage.

Among the participants, 28 (65.1%) had implants, with a predominance of dental prostheses (11; 25.6%), stents (3; 7.0%) and pacemakers, mitral valve prostheses and total hip arthroplasties (01; 2.3%). None of the patients used surgical drains.

The mean length of hospital stay was 27.58 days (SD = 20.67), ranging from 4 to 85 days. A total of 28 patients (65.1%) were admitted to the inpatient ward prior to coronary artery bypass grafting, with a mean stay of 19.36 days (SD = 14.45), ranging from 1 to 78 days.

The mean operating room temperature was 19.76°C (SD = 1.03), with a minimum of 17.1°C and a maximum of 22.1°C. The mean surgical duration was 3.64 h (SD = 0.83), ranging from 1 h and 55 min to 6 h. The mean duration of anaesthesia was 5.05 h (SD = 1.00), ranging from 3 h and 5 min to 7 h. All patients received general anaesthesia (43; 100.0%).

The majority of patients (41; 95.3%) underwent CPB during the surgical procedure. The mean CPB duration was 68 min (SD = 21.40), ranging from 35 to 127 min. All patients who underwent CPB experienced hypothermia (41; 95.3%). The mean duration of hypothermia during CPB was 49 min (SD = 17.16), with a minimum of 20 min and a maximum of 100 min.

Among the laboratory tests performed, serum levels of platelets, haemoglobin and haematocrit were collected in both the preoperative (pre‐op) and intraoperative (intra‐op) periods. The mean platelet count in the pre‐op period was 214.16 × 10^3^/μL (SD = 76.17), ranging from 117 to 486 × 10^3^/μL. In the intra‐op period, the mean decreased to 151.50 × 10^3^/μL (SD = 49.59), ranging from 34 to 253 × 10^3^/μL.

The mean haemoglobin level in the pre‐op period was 13.81 g/dL (SD = 5.34), with a range of 8.4 to 40.2 g/dL. During the intra‐op period, the mean haemoglobin also decreased to 10.21 g/dL (SD = 4.09), ranging from 3.7 to 28.3 g/dL. The mean haematocrit value in the pre‐op period was 34.95% (SD = 6.78), with a minimum of 10.5% and a maximum of 42.6%. In the intra‐op period, the mean haematocrit decreased to 26.67% (SD = 6.05), ranging from 8.5% to 38.5%.

Table 2 presents the variables related to the surgical safety checklist for patient safety and SSI prevention. The mean adherence score to the recommended practices was 71.18% (SD = 3.15), with a median of 70.58%, a minimum of 64.71% and a maximum of 80.39%. Individual adherence to checklist items ranged from 2.3% to 100.0%.

**TABLE 2:** Distribution of patients undergoing coronary artery bypass grafting (*N* = 43) according to items present in the Perioperative Surgical Safety Checklist (Roscani et al. 2015; Uberlândia, MG, Brazil, 2022–2023).

Admission to the surgical centre							
	Yes		No		Not applicable		
	*N* ^a^	%^b^	*N* ^a^	%^b^	*N* ^a^	%^b^	
Identification wristband present	43	100	—	—	—	—	
Preoperative bath	42	97.7	01	2.3	—	—	
Use of antibiotics in the last 24 h	02	4.7	41	95.3	—	—	
Under specific precautions	09	20.9	34	79.1	—	—	
Surgical site marked	—	—	43	100	—	—	
Respondent states which procedure will be performed	40	93.0	03	7.0	—	—	
Respondent confirms the surgical site location	41	95.3	02	4.7	—	—	
Known or declared allergy	05	11.6	38	88.4	—	—	
Pre‐anaesthetic evaluation form present	42	97.7	01	2.3	—	—	
Informed anaesthetic consent present	43	100	—	—	—	—	
Informed surgical consent present	43	100	—	—	—	—	
Hair removal performed	36	83.7	03	7.0	04	9.3	
Patient identification label in the medical record	43	100	—	—	—	—	
Presence of invasive devices	41	95.3	02	4.7	—	—	
Body temperature between 36°C and 36.5°C	17	39.5	26	60.5	—	—	
Before anesthesia induction and surgical field setup							
Patient's name and registration number verified	43	100	—	—	—	—	
Anesthesia cart tested and functioning	43	100	—	—	—	—	
Vital signs monitoring installed and functioning	43	100	—	—	—	—	
Difficult airway/risk of bronchoaspiration	09	20.9	34	79.1	—	—	
Assistance equipment available	43	100	—	—	—	—	
Considerable risk of blood loss (> 500 mL)	43	100	—	—	—	—	
Blood reserve confirmed	42	97.7	01	2.3	—	—	
All necessary materials and supplies present	41	95.3	02	4.7	—	—	
Validation and expiration date of sterilization of surgical instruments checked	43	100	—	—	—	—	
Scalpel blade positioned	43	100	—	—	—	—	
Patient positioned to avoid injuries	43	100	—	—	—	—	
Essential diagnostic images visible	42	97.7	01	2.3	—	—	
Surgical site antisepsis performed	43	100	—	—	—	—	
Blood glucose less than 200 mg/dL	41	95.3	02	4.7	—	—	
Before surgical incision							
All team members introduce themselves by name and role	—	—		43	100	—	—
Patient, procedure and surgical site identification confirmed by team members	42	97.7		01	2.3	—	—
Critical events anticipated for the surgical procedure	04	9.3		39	90.7	—	—
Critical events anticipated for anaesthesia procedure	07	16.3		36	83.7	—	—
Critical events anticipated for nursing procedure	03	7.0		40	93.0	—	—
Prophylactic antibiotic administered within last 60 min	43	100		—	—	—	—
FiO_2_ increased in patients with normal lung function undergoing endotracheal intubation^c^	43	100		—	—	—	—
Before leaving the operating room							
Count of compresses and gauzes correct	—	—	—	—	43	100	
Count of instruments and needles correct	43	100	—	—	—	—	
Material collected (anatomopathological or other)	43	100	—	—	—	—	
Request properly identified	43	100	—	—	—	—	
Any problems with materials, equipment or instruments	09	20.9	34	79.1	—	—	
Patient presents any skin lesion related to positioning or operative act	01	2.3	42	97.7	—	—	
All wear cap, mask, gloves and gown correctly during the procedure	15	34.9	28	65.1	—	—	
Any specific recommendation for immediate postoperative period	01	2.3	42	97.7	—	—	
FiO_2_ increase after extubation^c^	—	—	43	100	—	—	
Before leaving the surgical center							
Identification wristband present	40	93.0	03	7.0	—	—	
Presence of invasive devices	43	100	—	—	—	—	
Transoperative and anesthetic record in the medical record	43	100	—	—	—	—	
Surgical description signed in the medical record	43	100	—	—	—	—	
Any specific postoperative recommendation	43	100	—	—	—	—	
Prolonged surgical antimicrobial prophylaxis in postoperative period	43	100	—	—	—	—	

^a^ *N* = Number.

^b^ % = Percentage.

^c^ FiO_2_ = Fraction of inspired oxygen.

Binomial logistic regression analysis, using the total adherence score as the predictor, revealed that each additional percentage point in adherence to preventive measures was associated with a 10% reduction in the odds of infection (OR = 0.90; 95% CI: 0.73–1.10; *p* = 0.30); this association was not statistically significant due to the limited sample size of the study. However, the finding was clinically relevant (10% reduction), reinforcing the importance of considering adherence to preventive measures as a factor in reducing infection risk. The correlation between length of hospital stay and total adherence score was weak and not significant (*r* = −0.04; *p* = 0.80).

Of the 43 study participants, 24 (55.8%) developed SSI, of whom 10 (23.3%) required hospital readmission. Three patients (7.0%) died: one on the second postoperative day due to distributive and cardiogenic shock and another on the third day due to cardiogenic and vasoplegic shock, making it impossible to assess the development of SSI in both cases. The third patient developed SSI and died on the 45th day due to pneumonia and bloodstream infection.

Among the 24 participants who developed SSI, the following infection sites were identified: 11 (25.6%) in the sternotomy incision, 10 (23.3%) in the right saphenectomy site, 4 (9.3%) in the left saphenectomy site and 1 (2.3%) in the left radial artery site, considering that two participants developed infections at more than one site. The most common symptoms reported were the following: purulent discharge (12; 27.9%), serous discharge (8; 18.6%), erythematous wound edges (7; 16.3%), wound dehiscence (4; 9.3%) and pain (4; 9.3%). The mean time to symptom onset was 16.75 days (SD = 9.94), ranging from 6 to 45 days.

All patients with SSI received antimicrobial therapy (24; 100.0%). The most frequently used agents were the following: piperacillin‐tazobactam (14; 32.6%), teicoplanin (12; 27.9%), ciprofloxacin (9; 20.9%), meropenem (7; 16.3%) and amoxicillin‐clavulanate (7; 16.3%), which were used in combination regimens. The mean duration of antimicrobial therapy was 20.88 days (SD = 19.89), with a minimum of 3 days and a maximum of 80 days.

Regarding wound characteristics, all patients who developed SSI had an infected surgical wound (24; 100.0%). Positive culture results were obtained in 11 (25.6%) of these patients. The most commonly isolated pathogens were *Staphylococcus aureus* (6; 14.0%), *Klebsiella pneumoniae* (3; 7.0%) and *Serratia marcescens* (2; 4.7%). Additional pathogens included *Citrobacter koseri* (1; 2.3%), *Enterobacter cloacae* (1; 2.3%), *Klebsiella aerogenes* (1; 2.3%) and *Staphylococcus epidermidis* (1; 2.3%). It is worth noting that five patients had more than one pathogen identified in their culture results.

## 4 Discussion

Patients undergoing this intervention typically present a clinical profile characterized by advanced age, smoking, high ASA scores, high blood pressure, diabetes, dyslipidemia, obesity and physical inactivity (Mello et al. 2019). These factors are associated with a higher prevalence of SSI, as they are recognized predictors of postoperative complications due to their correlation with the patient's overall health status and the presence of comorbidities (Bhat et al. 2024).

Prolonged hospital stay, associated with greater exposure to microorganisms and invasive procedures, as well as long‐duration surgeries with extended extracorporeal circulation time, related to tissue manipulation, exacerbated inflammatory response and increased risk of microbial contamination, constitute relevant factors for SSIs (Sulzgruber et al. 2020; Jamil et al. 2020). In the studied population, the coexistence of these factors may have contributed to the high incidence of SSIs observed, underscoring the influence of hospital and operative exposure time on infectious outcomes.

This study identified a high adherence to the surgical safety checklist. Some items with incomplete adherence may be attributed to institutional protocols. For example, contact or droplet precautions are maintained for all patients transferred from another unit or for those with symptoms similar to those of the flu. In addition, extubation is performed 24 h after surgery, which explains the lack of an increase in FiO_2_ after extubation before leaving the operating room. Other issues were related to failures in systematization, such as the lack of guidance on preoperative bathing and availability of materials, failure to confirm blood reservation, inadequate verification of materials and supplies before the procedure and the team's noncompliance with necessary measures, such as the improper use of Personal Protective Equipment (PPE).

Hypothermia and glycemic control in the perioperative period are well‐established risk factors for the development of SSI. In the present study, the occurrence of hypothermia in patients undergoing CPB, although intentional due to its protective function for the organs during cardiac arrest, requires careful monitoring and the systematic implementation of warming strategies, such as the use of thermal blankets and heating of intravenous fluids (Cazella et al. 2022), strategies that were incorporated into the observed clinical practice. On the other hand, blood glucose levels were generally maintained within the recommended range of 80–120 mg/dL (Lai et al. 2022), suggesting that other care‐related factors had a greater influence on infection occurrence.

Prophylactic antibiotic administration occurred within 60 min prior to surgical incision in all patients, in accordance with recommendations from the literature, which indicate a significant association between appropriate timing of perioperative antibiotic prophylaxis and lower SSI rates (Sommerstein et al. 2023; Morris et al. 2025).

Although the wound‐healing process is influenced by multiple factors, including adequate tissue oxygenation, increasing the inspired oxygen fraction (FiO_2_) to 80% represents only one of the strategies aimed at optimizing the metabolic environment for tissue repair. In the present study, increasing the oxygen concentration before the surgical incision may have helped meet the higher metabolic demand associated with healing and the local immune response; however, the high incidence of SSI observed suggests that this measure alone was insufficient to mitigate the impact of other relevant risk factors. This finding is consistent with the multifactorial nature of sternum wound complications and infectious outcomes following sternotomy (Gomes et al. 2022; Busani et al. 2021; Song et al. 2023).

The infection rate observed in the present study (55.8%) is higher than those reported in the literature, which range from 8.4% to 15.2% (Andrade et al. 2019; Santos et al. 2018). Evaluations of a Brazilian state hospital network have demonstrated higher SSI rates in institutions located farther from the state capital and in public hospitals, suggesting that geographic and institutional factors may influence infectious outcomes in surgical care (Carvalho et al. 2021). These findings are consistent with the elevated SSI rate observed in the present study and support the notion that the contextual characteristics of healthcare services may contribute to infection risk, alongside patient‐related and perioperative factors.

In the present study, purulent drainage was the most frequently identified clinical sign among SSI cases in patients undergoing CABG, a finding consistent with previously reported literature (Pereira et al. 2023). The observed microbiological profile, with a predominance of *S. aureus* , also aligns with studies evaluating SSI in cardiac surgery (Enginoev et al. 2022; Tanamas et al. 2025). The prevalence of this pathogen, commonly associated with the patient's own skin flora or transmitted through contact during healthcare, underscores the importance of preventive strategies, such as preoperative decolonization and strict adherence to infection control practices, including the proper use of personal protective equipment.

Although the overall adherence score to preventive measures was relatively high, persistent failures at critical stages of care may have limited the effectiveness of prevention strategies, as reflected in the observed SSI rates. These findings are supported by the study by Stone et al. (2026), which showed that the absence of deep SSIs after CABG was associated with the rigorous implementation of standardized perioperative practices and the maintenance of high sterility standards, indicating that SSI prevention depends on the complete and consistent application of recommended interventions, with partial adherence potentially being insufficient to ensure patient safety.

In this context, the adoption of care bundles, composed of standardized and evidence‐based interventions, has been associated with a reduction in SSIs in patients undergoing CABG. These bundles include measures such as preoperative bathing with chlorhexidine gluconate, nasal decolonization, intraoperative antibiotic prophylaxis and perioperative glycemic control, aimed at reducing clinical practice variability and ensuring the full implementation of preventive barriers (Stone et al. 2026). The literature suggests that the consistent application of these sets of interventions is associated with lower SSI rates, especially when there is systematic implementation and training of multidisciplinary teams (Dhandapani et al. 2024). Thus, institutional adoption of bundles, combined with ongoing engagement of multidisciplinary teams, constitutes a central component for the effectiveness of prevention strategies and the sustained reduction of SSI rates in the context of CABG (Cussotto et al. 2025; Stone et al. 2026).

A potential limitation of this study is the small sample size, which limits the ability to perform more robust analyses of associations or to explore risk factors for SSI in greater depth. In this context, the present investigation should be considered preliminary and exploratory, highlighting the need for future studies with larger samples.

## 5 Conclusion

The results of this study demonstrated a relatively high level of adherence to preventive measures; however, persistent gaps in clinical practice were identified, such as the inadequate use of PPE. The high incidence of SSI observed (55.8%) underscores the urgent and early need to implement preventive strategies in CABG, starting at hospital admission and extending through the preoperative, intraoperative, postoperative and postdischarge phases of care.

The findings have important implications for nursing practice. Nurses play a strategic role in implementing and monitoring preventive measures throughout the perioperative period, as well as in managing quality improvement programs, reinforcing adherence to preventive practices, educating patients and families and identifying early signs of complications. Strengthening nursing‐led interventions to prevent SSIs can improve patient safety, reduce hospital readmissions and lower healthcare costs associated with infectious complications. In addition, the results highlight the importance of incorporating this topic into nursing education, expanding continuing education programs and implementing institutional protocols to prevent SSIs. Future research is needed to investigate factors influencing adherence to preventive measures and to evaluate nursing‐led intervention strategies that promote increased adherence to recommendations and improve clinical outcomes in patients undergoing cardiac surgery.

## Author Contributions

All authors designed the study. C.V.G. and G.L.V.S. collected the data. Data analysis and interpretation were performed by C.V.G., G.L.V.S., C.M.R. and M.B.G.R. C.V.G., G.L.V.S., A.G.P. and M.B.G.R. drafted the manuscript, while C.M.R. and V.N.F. critically revised it. All authors read and approved the final version for submission.

## Funding

The authors have nothing to report.

## Ethics Statement

All participants signed the Informed Consent Form and the privacy and confidentiality of the subjects, and the confidential data involved in the research were maintained. Ethical approval was obtained from the Research Ethics Committee of the Federal University of Uberlândia, Brazil (reference number: 5.511.734).

## Conflicts of Interest

The authors declare no conflicts of interest.

## Data Availability

The data that support the findings of this study are available from the corresponding author upon reasonable request.
